# Supplementary material for: Water-oriented magnetic anisotropy transition
Source: Nat Commun. 2021 May 12;12:2738. doi: 10.1038/s41467-021-23057-4 (PMC8115317; doi:10.1038/s41467-021-23057-4)
Supplement: Supplementary file 1 — Supplementary Information [file 41467_2021_23057_MOESM1_ESM.pdf]

Supplementary Information for

Water-oriented magnetic anisotropy transition

Su et al.

Correspondence to: sato@cm.kyushu-u.ac.jp

Supplementary Methods

All the reagents were obtained from commercial suppliers and were used without further purification. The ligands 2,6-bis(3-methylpyrazol-1-yl)pyrazine (mprpz) and deuterated mprpz (mprpz-d_{12}) were synthesized according to previously published methods with minor modifications[1,2].

Synthesis of mprpz ligand. To a suspension of 3-methylpyrazole (8.0 g) in DMF (200 ml) was added NaH (6.0 g, 60 % dispersion in paraffin liquid) washed with hexane and the mixture stirred under N_2 at room temperature for 1 hour. Then 2,6-dichloropyrazine (7.2 g) was added and the mixture was heated to 100 °C under N_2 and stirred for 16 hours before cooling to the room temperature. The suspension was poured into cold water slowly and the white solid was obtained. After filtering, washing with water, and drying, white powder was obtained in 62 % yield. ^{1}H NMR (400 MHz, $CDCl_3$): δ 9.08 (s, 1H, pyrazine), 8.36 (d, 1H, pyrazole), 6.31 (d, 1H, pyrazole), 2.39 (s, 3H, methyl).

Synthesis of mprpz-d_{12} ligand. A mixture of mprpz (1.0 mmol) and 10 % Pd/C (10 wt % of the substrate) in D_2O (10 mL) was added to the reactor (20 ml) under H_2 atmosphere and then kept at 175 °C for 24 hours. After cooling, the reaction mixture was extracted with ethyl acetate (3×20 mL). The combined organic phases were washed with H_2O (2×10 mL), dried over $NaSO_4$, and evaporated under reduced pressure to obtain a powder sample. Then recrystallized with n-hexane to obtain white needle-like crystals. ^{1}H NMR (400 MHz, $CDCl_3$): δ 9.08 (s, 1H, pyrazine), 8.36 (d, 1H, pyrazole), 6.31 (d, 1H, pyrazole), 2.39 (s, 3H, methyl). The average deuteration ratio is close to 95% from ^{1}H NMR data.

Reversibility of the structural transition of complex 1. The reversibility of the structural transition of complex **1** was determined from variable-temperature single-crystal XRD measurements. The unit cell was first determined at 190 K. The temperature was then decreased to 140 and 105 K, and the unit cell was determined again. Thereafter, the temperature was increased back to 140 and 190 K, and the unit cell was determined once again. Unit cell parameters at 190 K (initial): a = 8.2032(2) Å, b = 10.0546(3) Å, c = 10.9638(3) Å, α = 87.088(2)°, β = 84.153(2)°, γ = 68.400(3)°; at 140 K (cooling): a = 10.3473(3) Å, b = 10.7151(3) Å, c = 15.3271(4) Å, α = 95.476(2)°, β = 102.237(2)°, γ = 93.983(2)°; at 105 K: a = 8.2628(3) Å, b = 10.2018(5) Å, c = 10.6253(5) Å, α = 88.087(4)°, β = 83.066(4)°, γ = 67.211(4)°; at 140 K (heating): a = 10.3470(3) Å, b = 10.7135(3) Å, c = 15.3180(4) Å, α = 95.469(2)°, β = 102.189(2)°, γ = 93.946(3)°; at 190 K (final): a = 8.2012(3) Å, b = 10.0506(4) Å, c = 10.9607(4) Å, α = 87.120(3)°, β = 84.166(3)°, γ = 68.412(4)°.

Crystal structure of 1-d_{14} and 1 determined by powder neutron diffraction. The phases of $[Co(ONO_2)_2(D_2O)(\text{mprpz-}d_{12})]$ (**1-d_{14}**) at 71 K and 194 K were modeled on the structure of **1** from the single-crystal XRD at 70 K and 190 K, respectively. Both powder patterns were in excellent agreement, with the molecular structure allowed to relax with unequal constraints in terms of the Co–N(O), C(N)–C(N) and N–O bond lengths (d_{Co1-N1}, d_{Co1-N5} ≤ 2.158 Å; d_{Co1-N3} ≤ 2.117 Å; d_{Co1-O1} ≤ 2.121; d_{Co1-O4} ≤ 2.099 Å; $d_{Co1-O1W}$ ≥ 2.022 Å; d_{C8-N3} ≤ 1.33 Å; d_{N3-C5} ≥ 1.33 Å; d_{C8-C7}, d_{C5-C6} ≥ 1.39 Å; d_{C7-N6}, d_{C6-N6} ≤ 1.34 Å; 1.36 Å ≤ d_{N2-C4}, d_{N4-C9} ≤ 1.37 Å; 1.405 Å ≤ d_{C2-C3}, $d_{C10-C11}$ ≤ 1.415 Å; 1.35 Å ≤ d_{C3-C4}, d_{C9-C10} ≤ 1.36 Å; 1.365 Å ≤ d_{N1-N2}, d_{N4-N9} ≤ 1.375 Å; 1.325 Å ≤ d_{N1-C2}, d_{N5-C11} ≤ 1.335 Å; 1.39 Å ≤ d_{N2-C5}, d_{N4-C8} ≤ 1.40 Å; d_{N7-O1} ≥ 1.275 Å; d_{N7-O3} ≥ 1.24 Å; d_{C1-C2}, $d_{C11-C12}$ ≥ 1.49 Å at 71 K;

and 2.183 Å ≤ $d_{Co1–N1}$ ≤ 2.187 Å; 2.173 Å ≤ $d_{Co1–N5}$ ≤ 2.177 Å; 2.109 Å ≤ $d_{Co1–N3}$ ≤ 2.113 Å; $d_{Co1–O1}$ ≥ 2.098 Å; $d_{Co1–O4}$ ≥ 2.135 Å; $d_{Co1–O1W}$ ≤ 2.015 Å; $d_{C8–N3}$, $d_{C7–N6}$ ≥ 1.33 Å; $d_{C5–N3}$, $d_{C6–N6}$ ≤ 1.33 Å; $d_{C5–C6}$ ≥ 1.39 Å; $d_{C7–C8}$ ≤ 1.39 Å; 1.36 Å ≤ $d_{N2–C4}$, $d_{N4–C9}$ ≤ 1.37 Å; 1.405 Å ≤ $d_{C2–C3}$, $d_{C10–C11}$ ≤ 1.415 Å; 1.35 Å ≤ $d_{C3–C4}$, $d_{C9–C10}$ ≤ 1.36 Å; 1.365 Å ≤ $d_{N1–N2}$, $d_{N4–N9}$ ≤ 1.375 Å; 1.325 Å ≤ $d_{N1–C2}$, $d_{N5–C11}$ ≤ 1.335 Å; 1.39 Å ≤ $d_{N2–C5}$, $d_{N4–C8}$ ≤ 1.40 Å; $d_{N7–O1}$ ≥ 1.269 Å; $d_{N7–O2}$ ≥ 1.214 Å; $d_{N7–O3}$ ≥ 1.246 Å; $d_{N8–O4}$ ≥ 1.271 Å; $d_{N8–O6}$ ≥ 1.235 Å; 1.48 Å ≤ $d_{C1–C2}$, $d_{C11–C12}$ ≤ 1.50 Å at 194 K), angle restraints on the pyrazinyl, 3-methylpyrazolyl, and nitrate [angle (N6–C7–C8) ≤ 121.0°; angle (C9–C10–C11, C10–C9–N4, C2–C3–C4, C3–C4–N2) ≥ 106.5°; angle (C11–C12–N5) ≥ 121.0°; angle (O1–N7–O3) ≥ 119.0°; angle (H1A–C1–H1B, H1C–C1–H1B, H1A–C1–H1C, H12A–C12–H12B, H12C–C12–H12B, H12A–C12–H12C) ≥ 109.0° at 71 K; angle (C9–C10–C11, C10–C9–N4, C2–C3–C4, C3–C4–N2) ≥ 106.5°; angle (N3–C5–C6, N6–C7–C8) ≥ 120.0°; angle (N2–C5–C6) ≥ 125.0°; angle (N1–C2–C1) ≥ 121.0° at 194 K], and torsional angle restraints on the the pyrazinyl and 3-methylpyrazolyl [angle (C11–C10–C9–N4, C2–C3–C4–N2, C4–C3–C2–N1) ≤ 1.0°; angle (C8–C7–N6–C6, N3–C5–C6–N6) ≤ 3.0°; angle (C9–C10–C11–C12, C1–C2–C3–C4) ≥ 177.5° at 71 K; and angle (N3–C5–C6–N6) ≤ 3.0° at 194 K]. The B_{iso} parameters for non-deuterated atoms and deuterated atoms (except for water molecules) were constrained using $B_{iso}(D)/B_{iso}(non\text{-}D) = 1.5$ at 71 K ($B_{iso}(non\text{-}D) = B_{iso}(Co1)$). All atoms (except for water molecules) of the same type were constrained to ensure equivalent B_{iso} parameters at 194 K ($B_{iso}(C) = B_{iso}(C1)$, $B_{iso}(N) = B_{iso}(N1)$, $B_{iso}(O) = B_{iso}(O1)$, and $B_{iso}(D) = B_{iso}(D3A)$). From neutron scattering measurements, the locations of the D atoms in the water molecules were unambiguously determined for the structure and were freely refined, along with the location of O1W. The locations of the other D atoms were also clearly determined and freely refined. The results of the refinements are provided in cif files (CCDC 2020263-2020264) and summarized in table S4.

Determination of water orientation. To determine the unambiguous orientation of water, here, we use high-resolution single crystal diffraction (HRXRD) and powder neutron diffraction (PND). Generally, single-crystal neutron diffraction is the preferred method to accurately determine the position of hydrogen atoms. However, single crystal **1** cannot be analyzed in this way because it easily breaks after the first-order phase transition. The structure of **1** was analyzed with high-resolution single-crystal X-ray diffraction. The results show that the dihedral angle φ changes from 89.17° at 190 K to 68.00° at 70 K with almost the same variation (21.2°) as in the XRD measurements, while the angle φ_1 only changes by 0.06°. The bond lengths and angles between non-hydrogen atoms are almost unchanged compared with the XRD results (Supplementary Tables 1 and 2). However, there is still uncertainty about the orientation of water molecules because of the vibration or disorder of H atoms, which could be obtained from the different atom displacement parameters of the H atoms in water at Lp and Ip, especially for the 70 K (HRXRD) data. In addition, the structures of the deuterated analog [Co(ONO_2)$_2$(D_2O)(mprpz-d_{12})] (**1-d_{14}**) were analyzed using variable-temperature single crystal XRD (120, 150, and 190 K) and powder neutron diffraction (71 K and 194 K) (Supplementary Fig. 6). From the single crystal XRD data, the angle φ changes by 15.61° from 83.31° at 190 K to 67.70° at 120 K, while the angle φ_1 only changes by 0.23°. The neutron diffraction data show that the dihedral angle φ is related to the angle of the water molecule and changes from 88.50° at 194 K to 67.73° at 71 K, while the angle φ_1 only changes by 2.41° (Supplementary Table 1 and 5).

From four sets of data, the water reorientation in complex **1** is mainly attained by the rotation of water molecules around the Co–O bond after the structural transition. The variation of the angle φ

in complex **1** from HTp to LTp is described in the text with the result of HRXRD that is 21.2±0.2° (Supplementary Methods and Table 4). Herein, we include the average value and estimated deviation of the variation of the angle φ of 22±1° from data SCXRD-1 and SCXRD-1(hr). The difference between them lies within the experimental error bar.

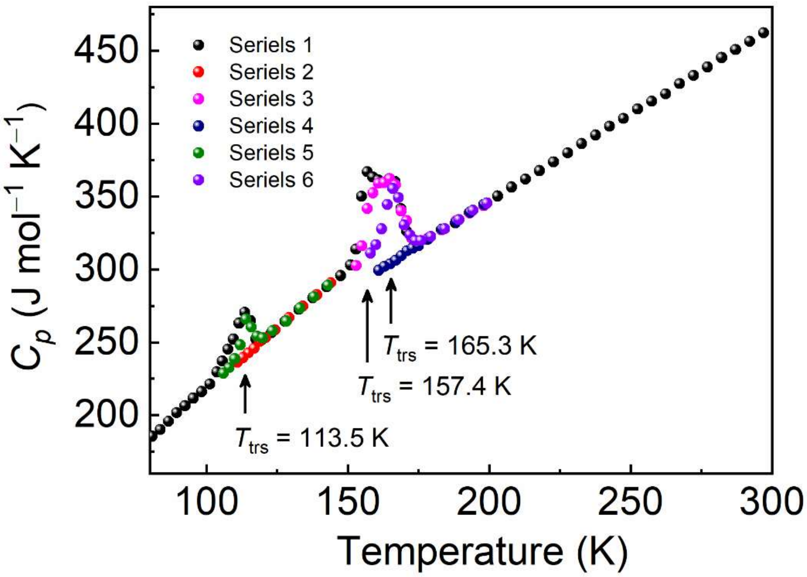

Supplementary Figure 1 Effect of thermal cycling on the heat capacity in crystal **1**. Over six cycles, the sample behaved in an identical way and almost no changes in the phase-transition temperatures were observed. The enthalpy and entropy transitions estimated from the heat capacity are ΔH = 249.6 ± 2.3 J mol^{-1} and ΔS = 2.242 ± 0.020 J K^{-1} mol^{-1}, respectively, for the phase transition at 113.5 K, and ΔH = 893.8 ± 6.6 J mol^{-1} and ΔS = 5.544 ± 0.041 J K^{-1} mol^{-1}, respectively, for the phase transition at 157.4 K.

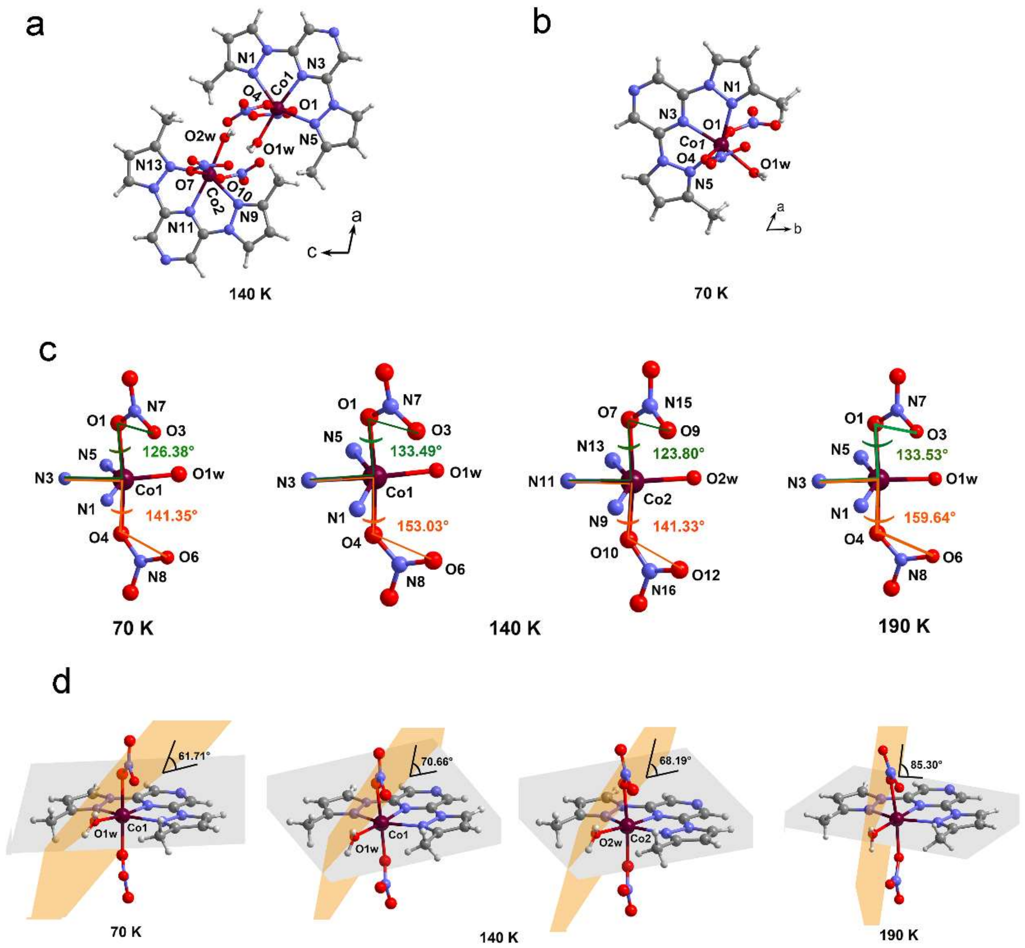

Supplementary Figure 2 Molecular structure and structure parameters of complex **1** at (a) 140 K and (b) 70 K. The cobalt centers in both states are six-coordinate, incorporating a ligand mprpz, two nitrates, and a water molecule. There are two crystallographically independent molecules in the asymmetric unit at 140 K. (c) Changes in the dihedral angles N3–Co1–O1–O3 (ψ) and N3–Co1–O4–O6 (ω) at LTp, Ip, and HTp, where both the dihedral angles ψ and ω increase by 7.15° and 18.29° from 70 to 190 K, respectively, through the rotations of the nitrates. (d) Variations in the dihedral angle, φ, between the molecular plane and the plane of the coordinated water molecule at LTp, Ip, and HTp. The atoms are shown as ball-and-stick representations. Maroon, Co; gray, C; blue, N; red, O; and light gray, H.

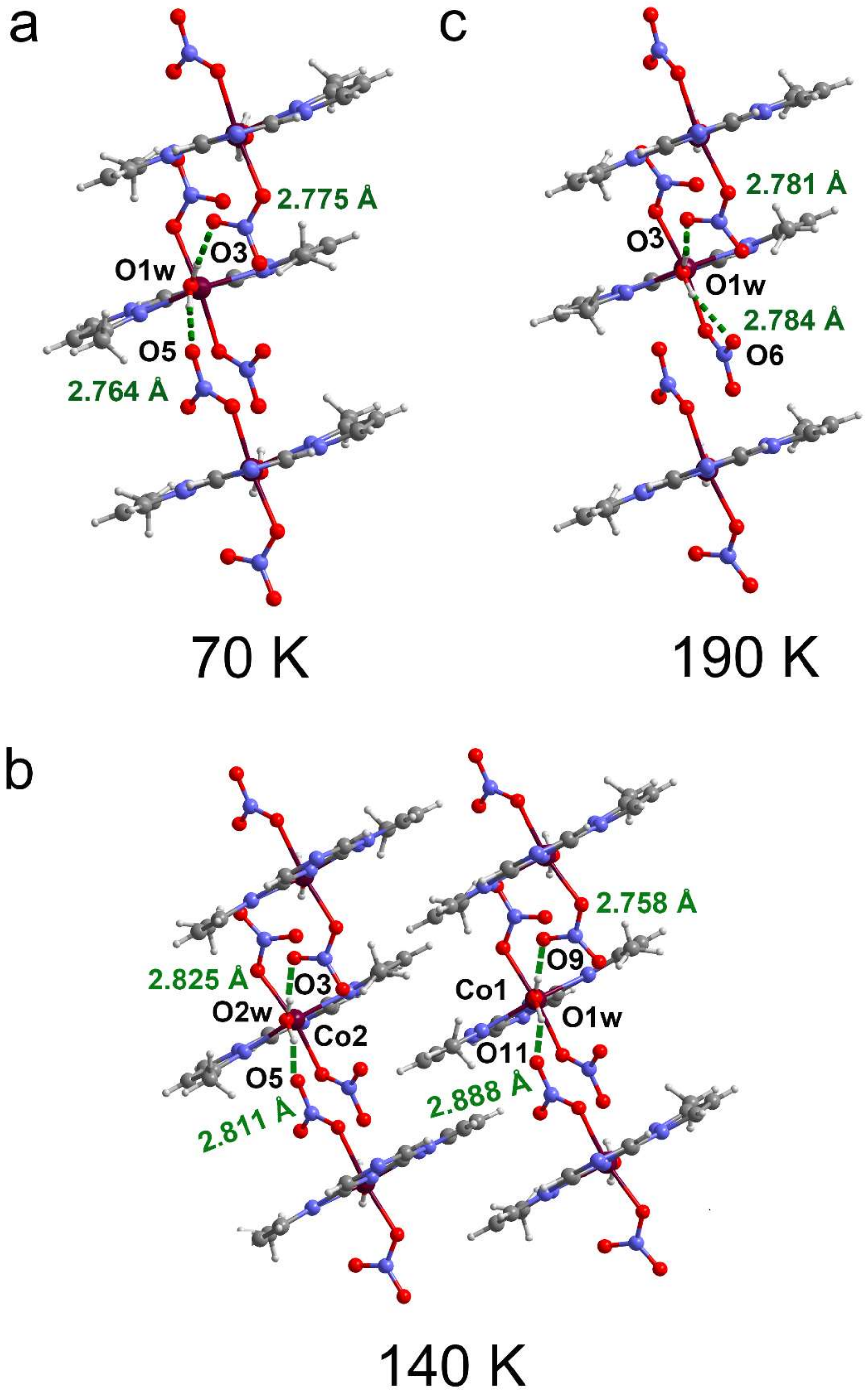

Supplementary Figure 3 Hydrogen bonding and the orientation of the water, where the coordinated water is surrounded by four nitrates. The donor water molecule forms four hydrogen bonds with the nitrates. Two stronger hydrogen bonds, which determine the water orientation, are shown for (a) LTp, (b) Ip, and (c) HTp as green dotted lines. Maroon, Co; gray, C; blue, N; red, O; and light gray, H.

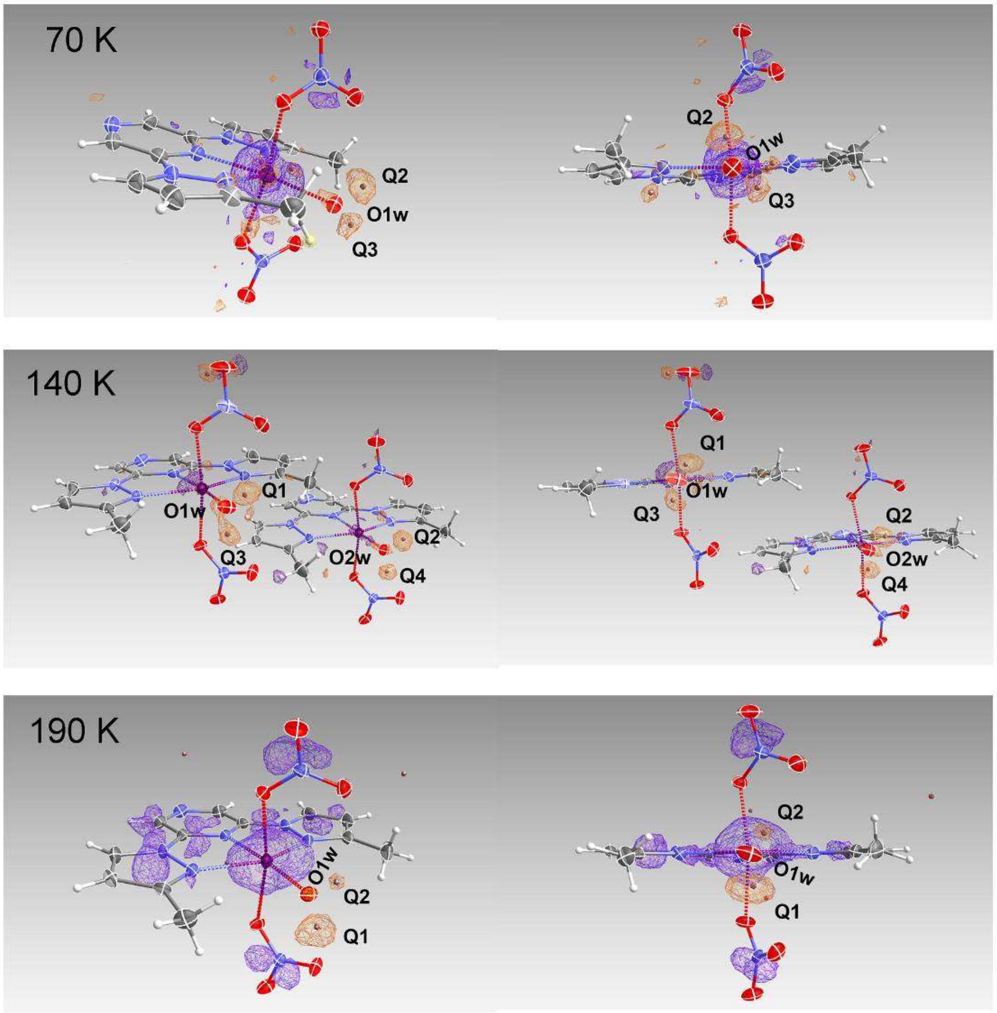

Supplementary Figure 4 Electron density (F_o–F_c) map of complex **1**. (Top) Electron density (F_o–F_c) map in the LTp structure at the 2.7 σ isocontour level. The electron densities of the Q2 and Q3 peaks nearest to the O1w atom are up to 5.9 and 5.6 σ, respectively. (Middle) Electron density (F_o–F_c) map in the Ip structure at the 4.2 σ isocontour level. The electron densities of the Q1 and Q3 peaks nearest to the O1w atom are up to 8.9 and 7.8 σ, and those of the Q2 and Q4 peaks nearest to the O2w atom are up to 8.6 and 6.8 σ, respectively. (Bottom) Electron density (F_o–F_c) map in the HTp structure at the 2.1 σ isocontour level. The electron densities of the Q1 and Q3 peaks nearest to the O1w atom are up to 3.4 and 2.9 σ, respectively.

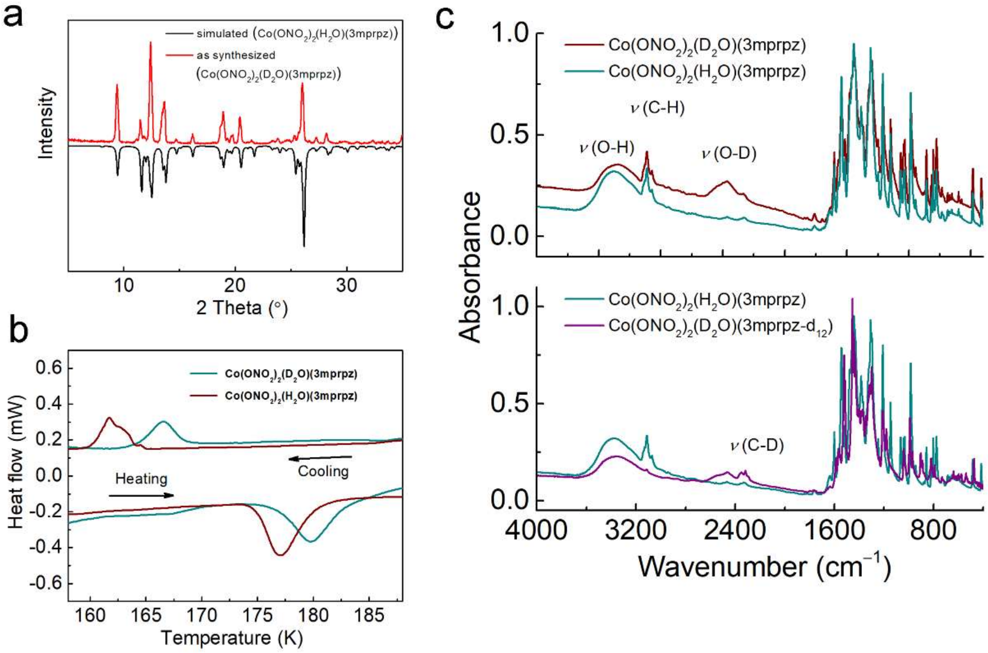

Supplementary Figure 5 PXRD patterns, DSC curves, and IR spectra of complex **1** and the deuterated sample. (a) PXRD patterns of complex **1** and the partially deuterated sample **1-*d*$_2$**. The experimental pattern of the deuterated sample is in good agreement with that of complex **1**, which suggests that **1-*d*$_2$** is an analog of complex **1**. (b) DSC measurements indicate that the phase transition temperature of the deuterated sample is slightly higher (by *ca*. 3 K) than that of complex **1**. (c) The IR spectra show that the nearly all hydrogen atoms in the ligand have been replaced by deuterium atoms. The degree of deuteration of the coordinated water molecules is estimated to be 28%, as determined by the decrease in the intensity of the O–H band in the IR spectrum using the C−H band intensity as a reference peak.

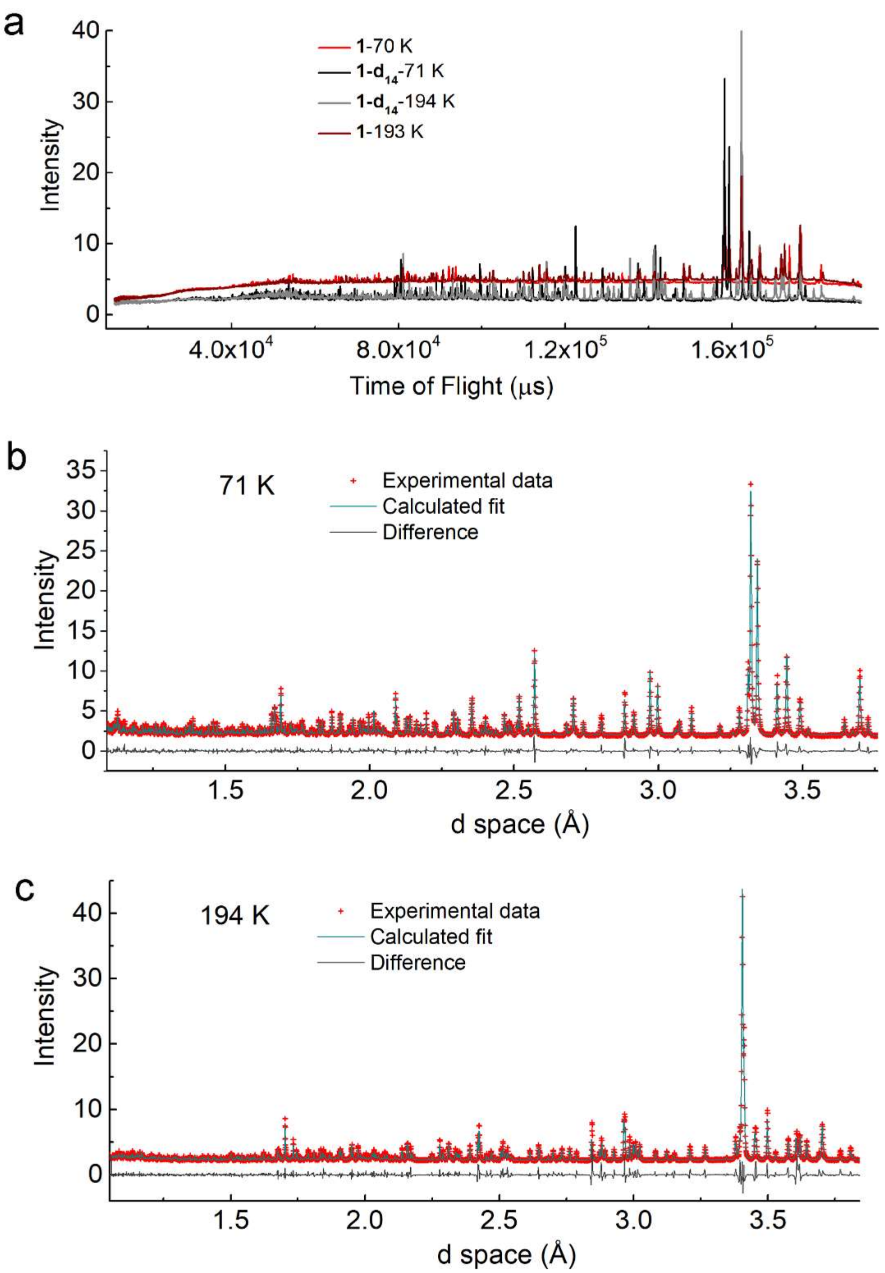

Supplementary Figure 6 Variable-temperature neutron powder diffraction data. (a) The profiles of data for [Co(ONO$_2$)$_2$(H$_2$O)(mprpz)] (**1**) and [Co(ONO$_2$)$_2$(D$_2$O)(mprpz-*d*$_{12}$)] (**1-*d*$_{14}$**) measured at different temperatures. The non-deuterated sample was observed higher background level which comes from incoherent scattering of hydrogen than the deuterated one. The diffraction data of the deuterated and non-deuterated samples are nearly the same across the different temperatures, where the data can be observed to change significantly with temperature, which is consistent with the results of the single-crystal x-ray diffraction and thermal analysis measurements. Fitting results of data for [Co(ONO$_2$)$_2$(D$_2$O)(mprpz-*d*$_{12}$)] (**1-*d*$_{14}$**) measured at (b) 71 K, and (c) 194 K, showing the experimental data (red), calculated fit (cyan), and difference profile (black).

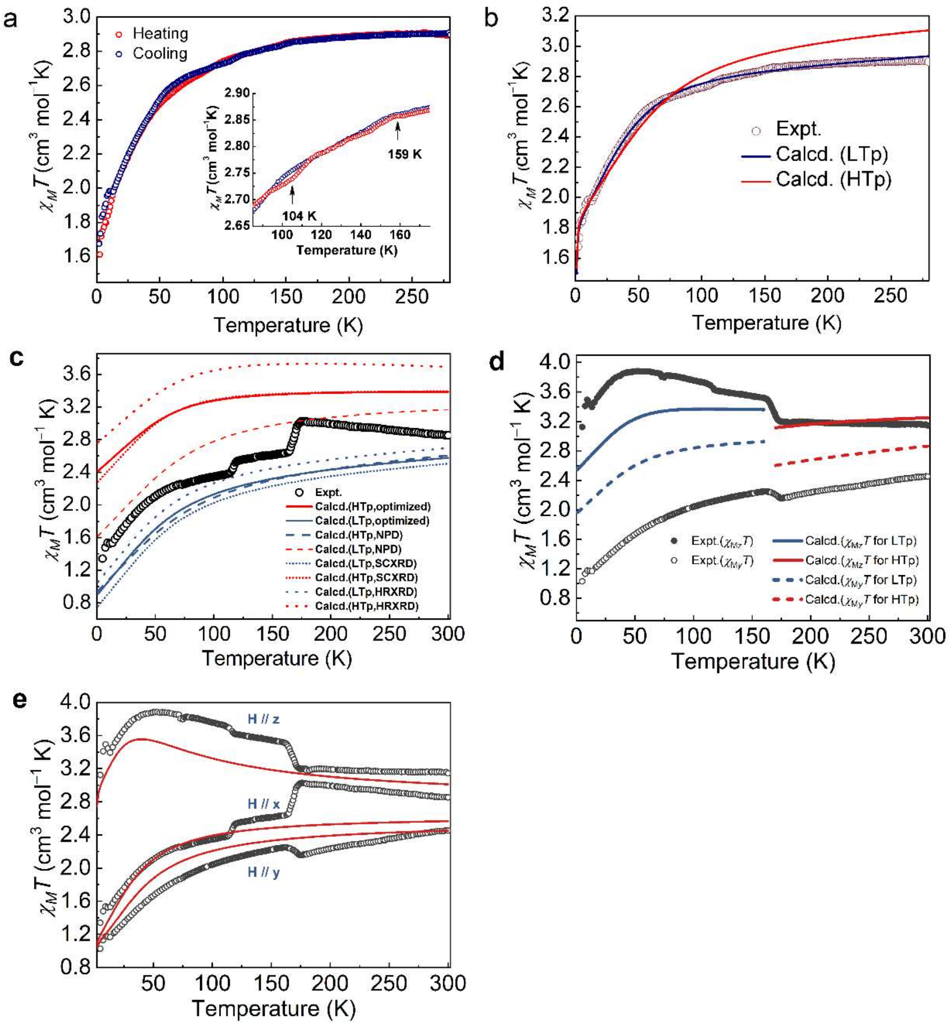

Supplementary Figure 7 The experimental and calculated $\chi_M T$ curves for a microcrystalline sample of **1**. (a) Variable-temperature $\chi_M T$ data for a microcrystalline sample of **1** was collected from 5 to 275 K under 2000 Oe in cooling (blue) and heating (red) modes. The irregular shapes of the curves may be because of structural fluctuations. (b) The calculated $\chi_M T$ values at LTp are in good agreement with the experimental data. In the high temperature phase (HTp) data, the calculated $\chi_M T$ values are slightly overestimated, which could be attributed to the contribution from vibronic coupling. (c) Experimental and *ab initio* calculated $\chi_{Mx} T$ curves in LTp and HTp. The changes in the calculated values after the phase transition are consistent with the experimental data. The solid line represents the calculation result of the molecular structure with optimized hydrogen atom position; the dash line represents the calculation result of the molecular structure from neutron powder diffraction (NPD); the short dot line represents the calculation result of the molecular structure from single crystal X-ray diffraction (SCXRD); and the dot line represents the calculation result of the molecular structure from high-resolution single crystal X-ray diffraction (HRXRD). (d) Experimental and *ab initio* calculated $\chi_{My} T$ and $\chi_{Mz} T$ curves in LTp and HTp. (e) Experimental (black cycles) and simulated (red lines) temperature-dependent anisotropic magnetic susceptibility. The anisotropic magnetic can be reproduced with the Hamiltonian parameters D, E, g_x, g_y and g_z obtained from HF-EPR analysis.

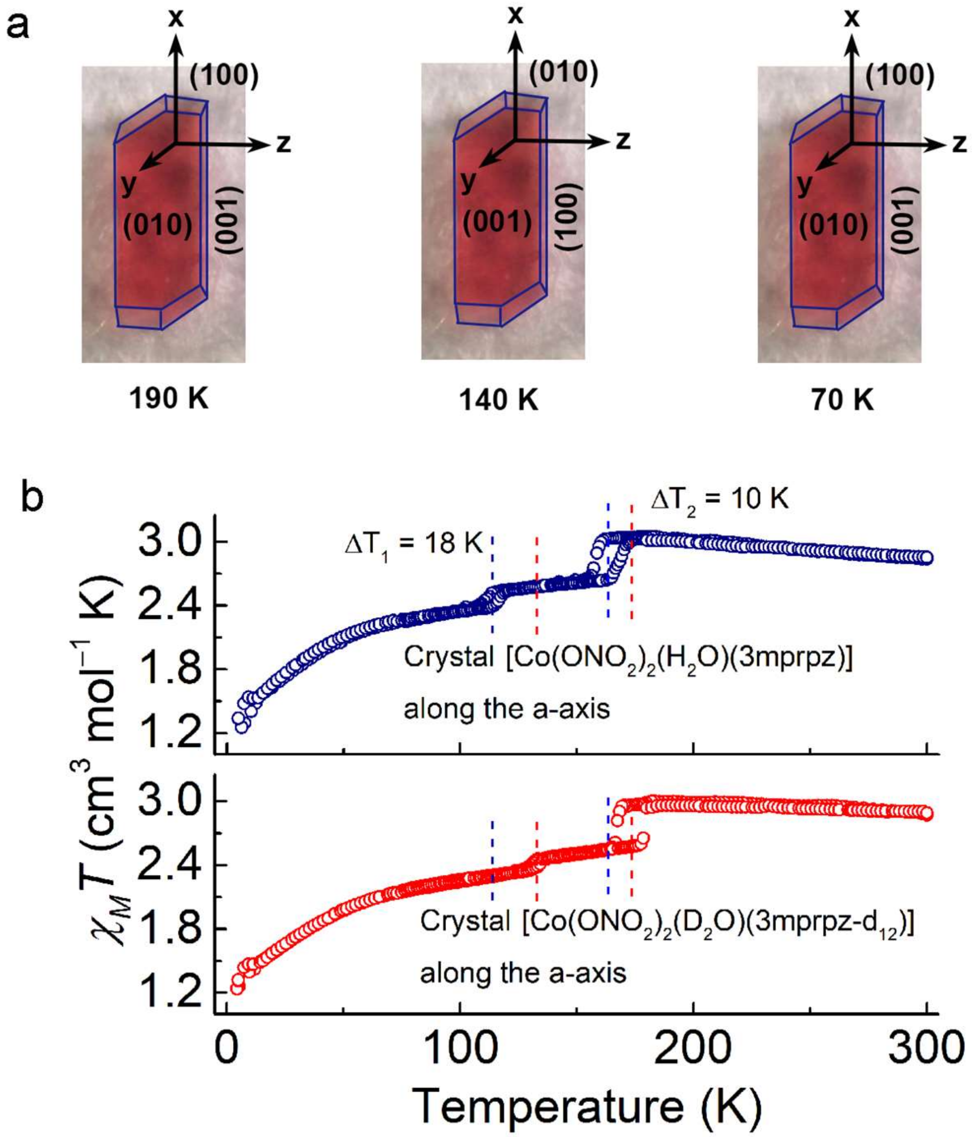

Supplementary Figure 8 (a) A schematic diagram showing the relationship between the *x*, *y*, and *z* orthogonal directions and the crystal faces of a single-crystal at HTp, Ip, and LTp. (b) Temperature dependence of $\chi_{Mx}T$ for the single crystals $[Co(ONO_2)_2(H_2O)(mprpz)]$ (**1**) and $[Co(ONO_2)_2(D_2O)(mprpz\text{-}d_{12})]$ (**1-*d*$_{14}$**). Both of them show similar changes in their temperature-dependent magnetic susceptibility along the *x*-direction (*a*-axis) with different phase transition temperatures. The transition temperatures increased significantly for **1-*d*$_{14}$**, which can be attributed to the stronger hydrogen bonding interactions present in the deuterated sample. The blue dotted lines represent the phase transition points of **1** and the red dotted lines represent the phase transition points of **1-*d*$_{14}$**.

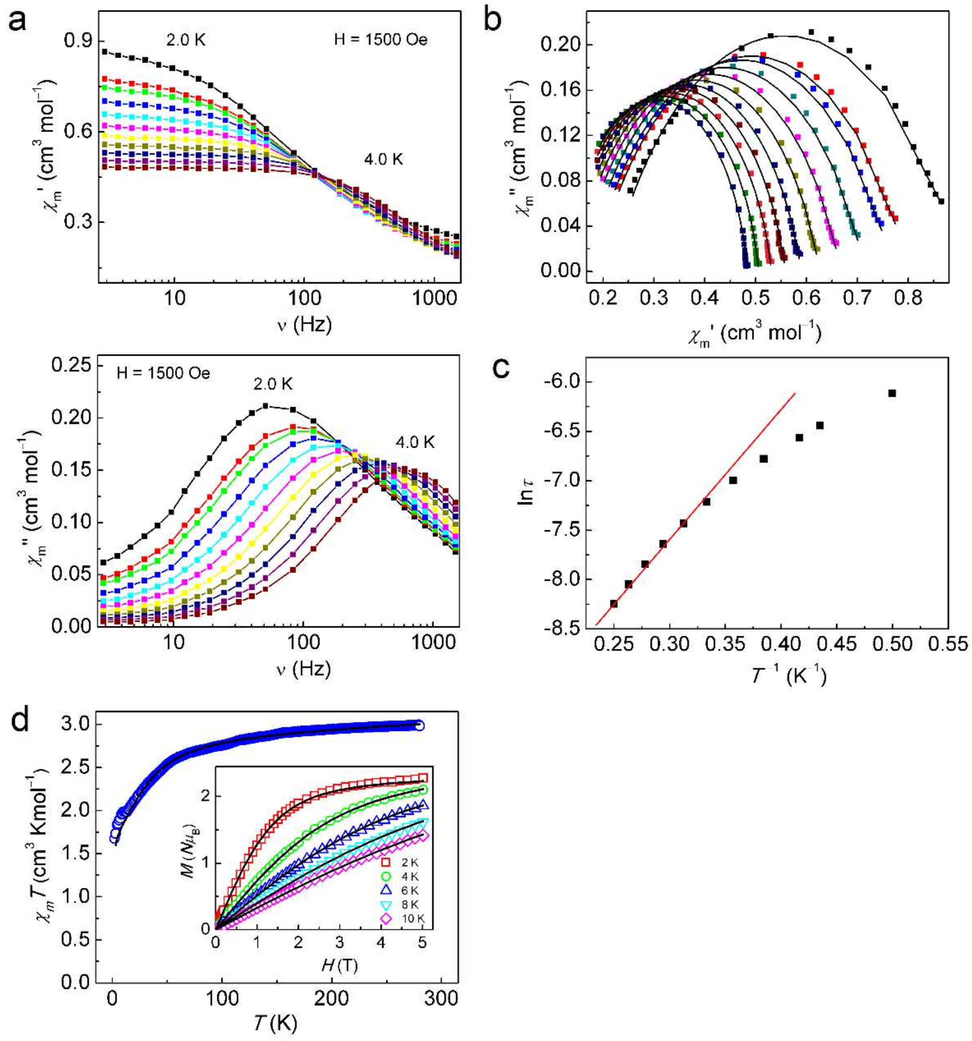

Supplementary Figure 9 Dynamic and static magnetic data. (a) Frequency dependence of the ac magnetic susceptibility collected over the temperature range of 2.0–4.0 K under an applied dc field of 1500 Oe. (b) Cole–Cole plots of χ'' versus χ' over a temperature range of 2.0−4.0 K under a 1500 Oe applied dc field. The solid lines represent the best fits of the experimental results according to a generalized Debye model. The calculated distribution coefficients, α, are within the range of 0.11–0.31 (2.0–4.0 K), indicating a relatively narrow distribution of relaxation times. (c) Arrhenius plot constructed using the ac data. The solid red line represents the linear fitting of the experimental data to the Arrhenius equation, $\tau = \tau_0 \exp(U_{\text{eff}}/k_B\text{T})$, where τ is the relaxation time, U_{eff} is the effective energy barrier for the reversal of the magnetization, τ_0 is the pre-exponential factor, and k_B is the Boltzmann constant. The U_{eff} values obtained from the linear fitting of the experimental data is 8.6 cm^{-1} ($\tau_0 = 1.2 \times 10^{-5}$ s). (d) Temperature dependence of $\chi_m T$ at 0.1 T. Inset: magnetization data at 2−10 K. The solid lines represent the theoretical fittings using the PHI program by the anisotropic spin Hamiltonian (with S = 3/2 and $g_{(X)} = g_{(Y)}$) as given in eq 1,[3] $H = D(\hat{S}_Z{}^2 - S(S+1)/3) + E(\hat{S}_X{}^2 - \hat{S}_Y{}^2) + \mu_B g\hat{S}\cdot\hat{H}$ (1). The best-fit parameters are $g_{(X)} = g_{(Y)} = 2.36$, $g_{(Z)}$ = 2.71, $D = 32.01\ \text{cm}^{-1}$, and $|E| = 0.28\ \text{cm}^{-1}$.

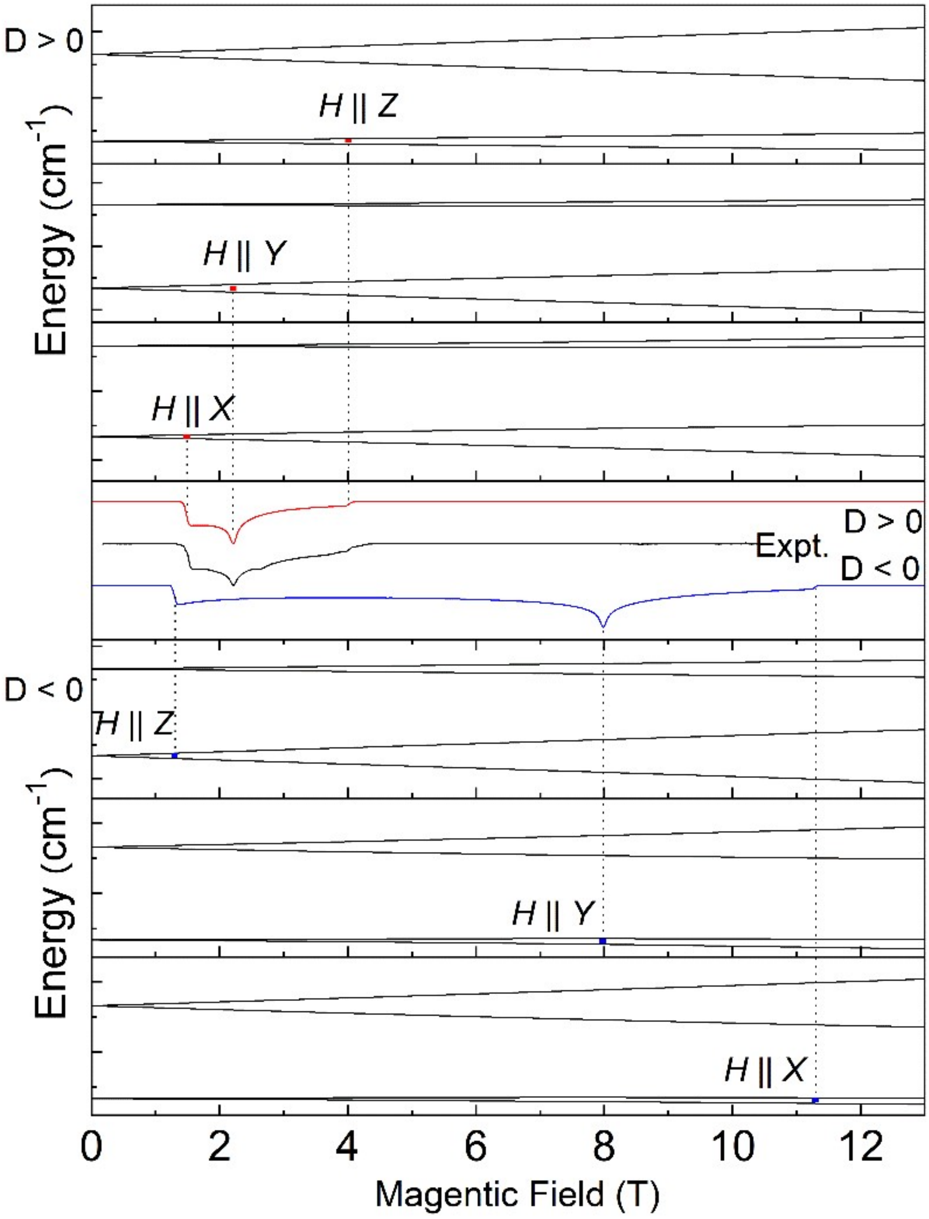

Supplementary Figure 10 HF-EPR spectra and energy levels as a function of magnetic field for the three canonical orientations of the field relative to the principal zfs axis at $T = 4.2$ K and $\nu = 120$ GHz. The spin Hamiltonian parameters used in simulations are: $S = 3/2$, an axial g-tensor ($g_{(X)} = g_{(Y)} = 2.45(2)$, $g_{(Z)} = 2.25(2)$), $|D| = 32.01$ cm^{-1}, and $|E| = 4.30(2)$ cm^{-1} ($E/D \sim 0.13$).

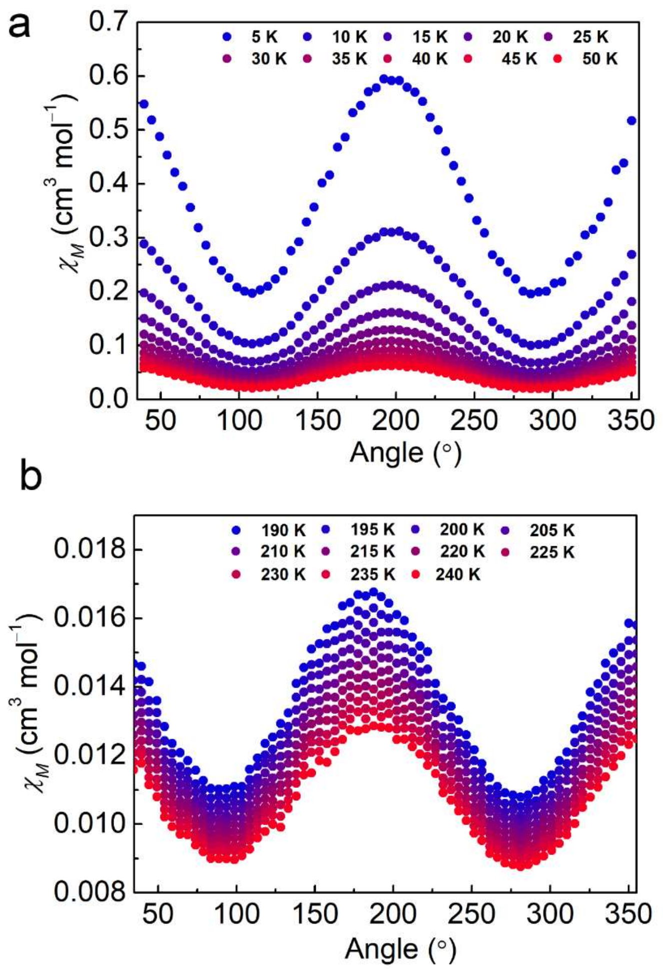

Supplementary Figure 11 Temperature dependence of the angular variation around the *x* axis of the molar magnetic susceptibility for the (a) LTp and (b) HTp structures.

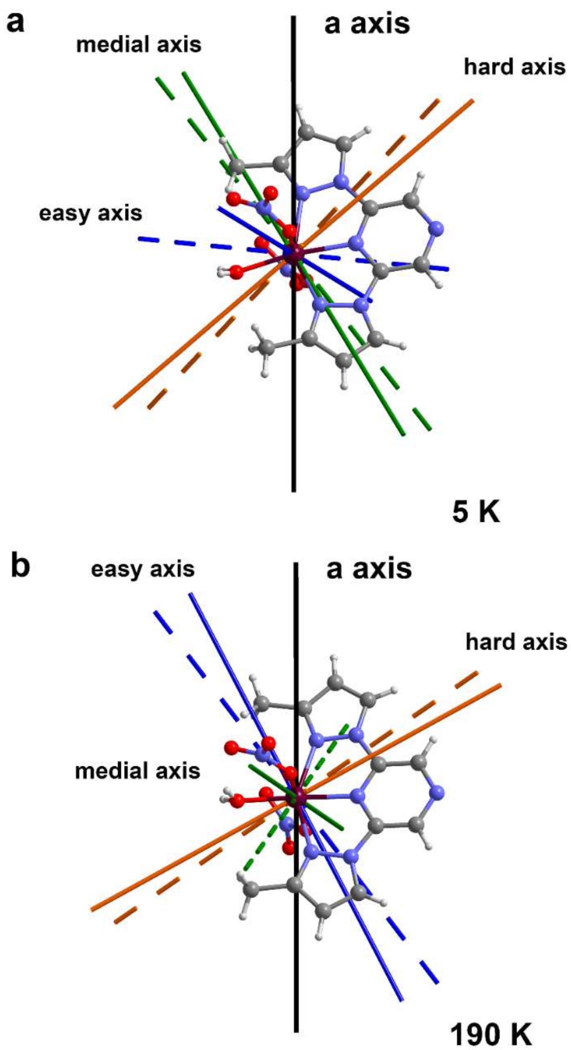

Supplementary Figure 12 Experimental (solid lines) and *ab initio* calculated (dashed lines) susceptibility tensors of the magnetization at 5 K and 190 K for **1**. (a) The angles of the deviations between the experimental values and the calculated data at 5 K are 21.1° for the hard axis, 8.6° for the medial axis, and 22.3° for the easy axis. (b) The angles of the deviations between the experimental values and the calculated data at 190 K are 15.5° for the hard axis, 25.4° for the medium axis, and 21.3° for the easy axis. Maroon, Co; gray, C; blue, N; red, O; and light gray, H.

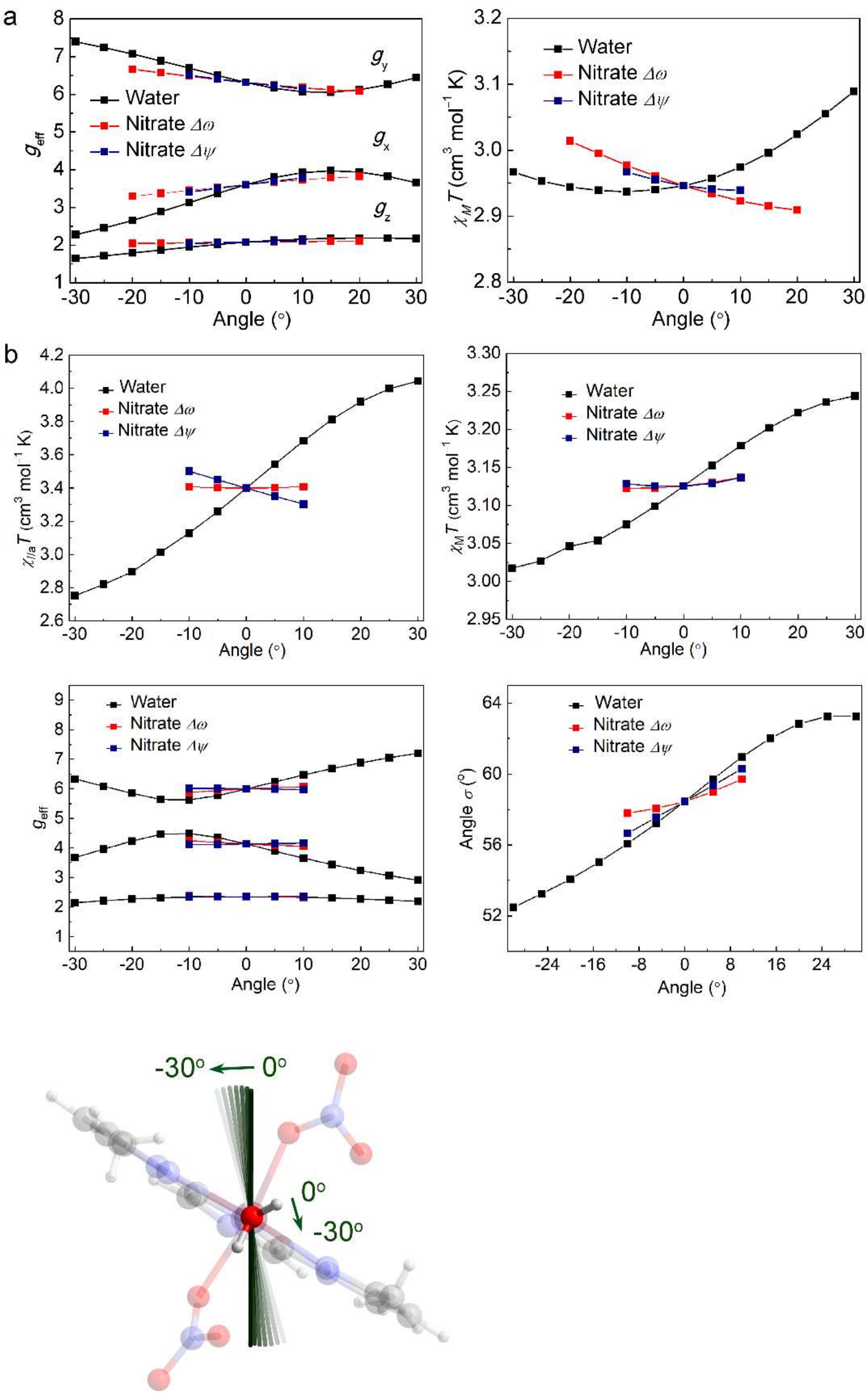

Supplementary Figure 13 (a) The effect of the g tensor values (S = 1/2) and changes in magnetic susceptibility according to the rotation angle for the two models in the LTp structure. (b) The changes of magnetic susceptibility along the a-axis, average magnetic susceptibility, effect of the g tensor values (S = 1/2), angle σ and magnetic hard axis according to the rotation angle for the two models in the HTp structure.

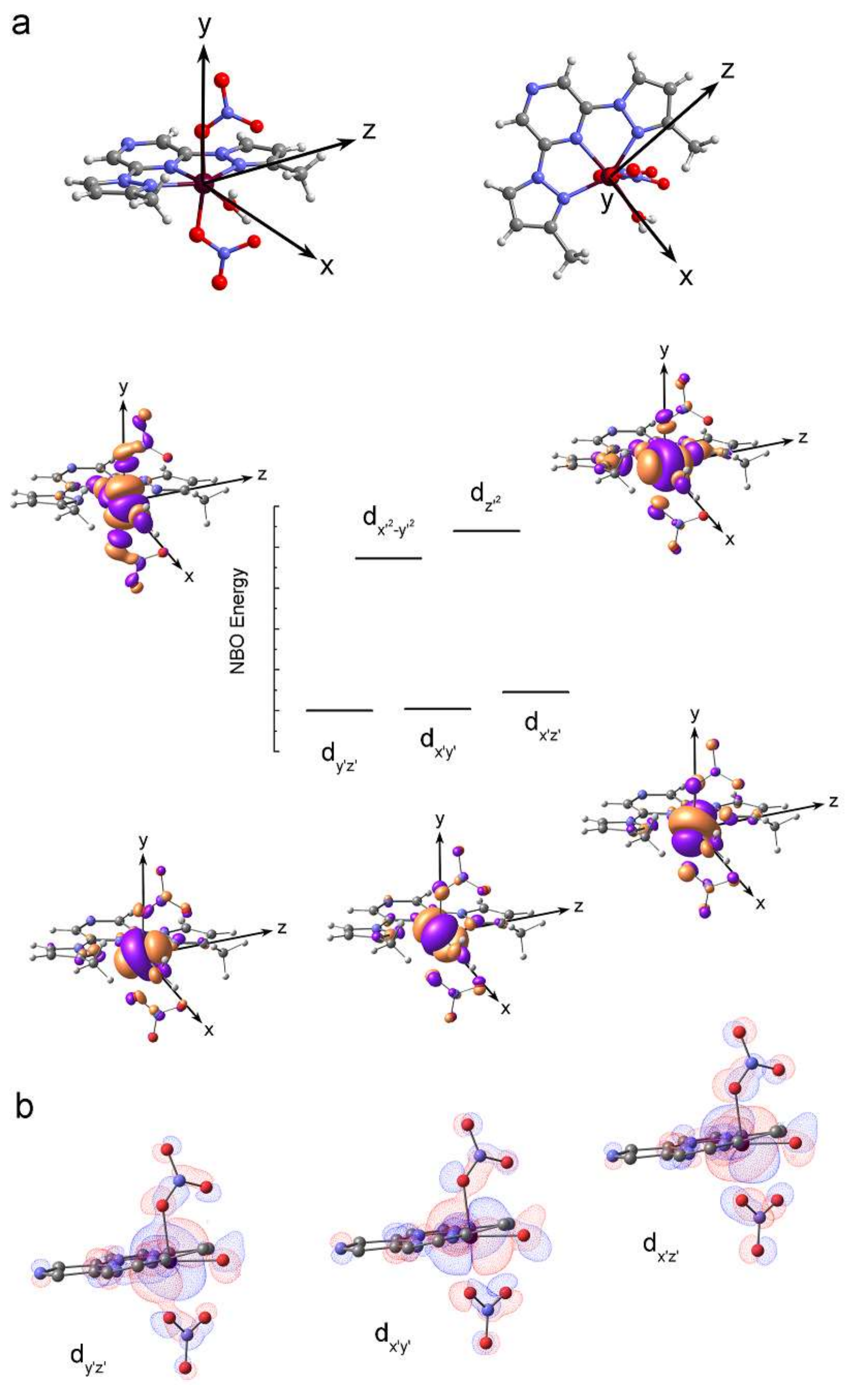

Supplementary Figure 14 The d orbital energies of the LTp. (a) Geometric structure and choice of coordinate axes, and *ab initio* (NEVPT2) ligand field d orbital energies. (b) In the occupied orbitals ($d_{y'z'}$, $d_{x'y'}$, and $d_{x'z'}$), the overlap between the d orbital centered on the cobalt and the p orbitals of the oxygen atoms from the water and nitrates. The contour value of the wave function is 0.005. The red color corresponds to the regions where the phase of the wave function is positive and the blue color corresponds to the regions where the phase of the wave function is negative. Maroon, Co; gray, C; blue, N; red, O; and light gray, H.

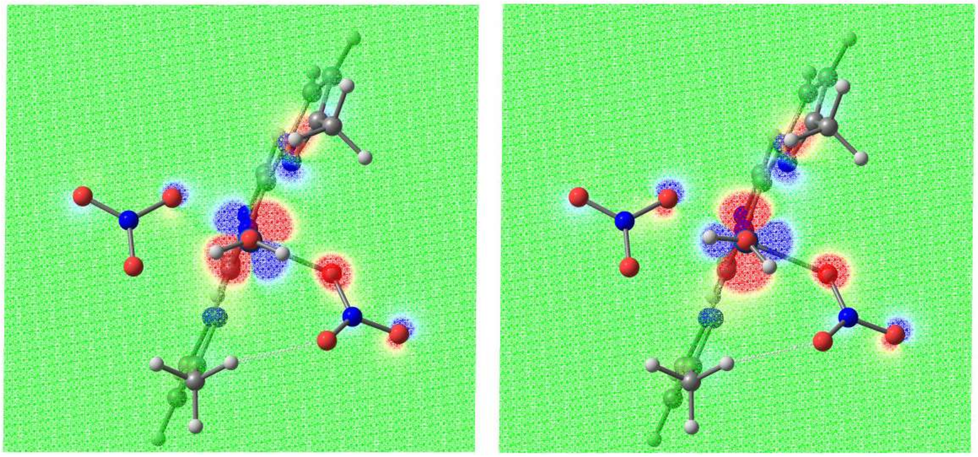

Supplementary Figure 15 The $d_{x'y'}$ orbital projected onto the plane passing the Co(II) center and perpendicular to the Co–O bond before (left) and after rotating 25° (right).

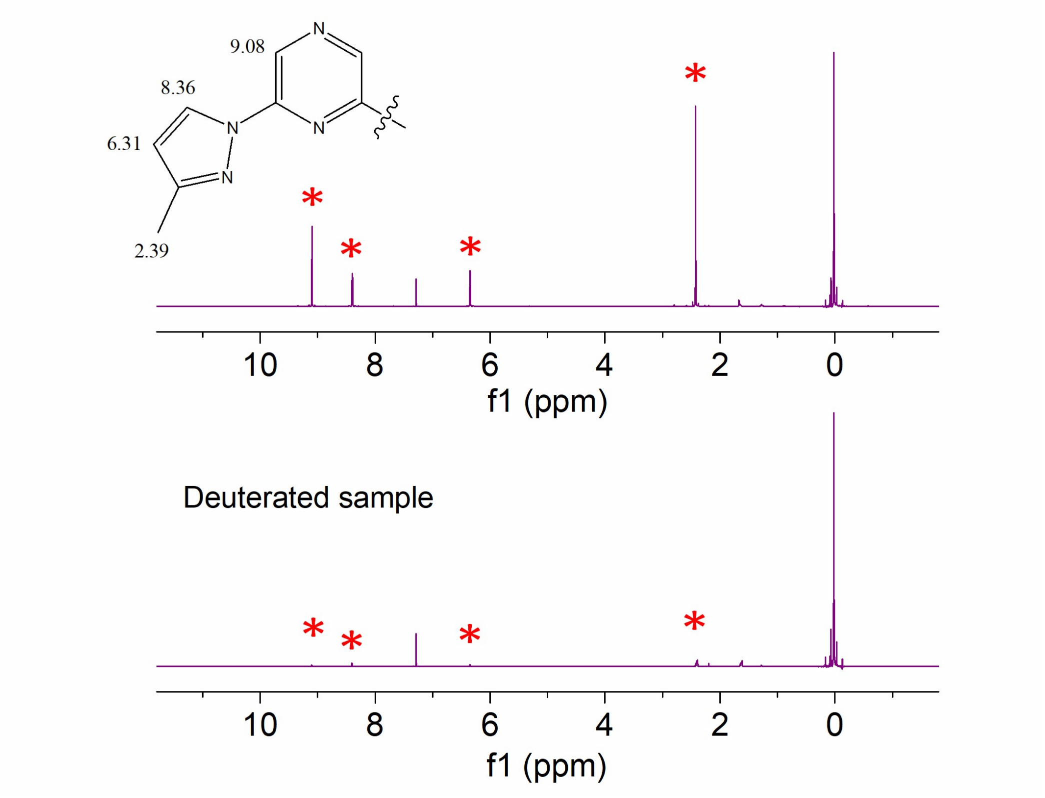

Supplementary Figure 16 ^{1}H NMR spectra of mprpz and deuterated mprpz ligands in $CDCl_3$.

Supplementary Table 1 Crystallographic parameters for the complexes **1** and **1-*d*$_{14}$** at different temperatures.

complex **1**			
	70 K	140 K	190 K
Formula	$C_{12}H_{14}CoN_8O_7$	$C_{12}H_{14}CoN_8O_7$	$C_{12}H_{14}CoN_8O_7$
Formula weight	441.24	441.24	441.24
Crystal system	Triclinic	Triclinic	Triclinic
Space group	*P*-1	*P*-1	*P*-1
a (Å)	8.2667(11)	10.360(2)	8.2322(8)
b (Å)	10.2094(15)	10.786(2)	10.0593(10)
c (Å)	10.6408(19)	15.361(3)	10.9956(10)
α (deg)	88.033(12)	95.45(3)	87.111(6)
β (deg)	83.097(12)	102.13(3)	84.082(6)
γ (deg)	67.145(9)	93.79(3)	68.301(5)
V (Å^3)	821.5(2)	1664.0(6)	841.45(14)
Z	2	4	2
D_{calcd} (g cm^{-3})	1.784	1.761	1.742
F(000)	450	900	450
R(int)	0.0910	0.0279	0.0379
GOF on F^2	1.191	0.941	1.107
R_1[a] [$I > 2\sigma(I)$]	0.0576	0.0307	0.0305
ωR_2[b](all data)	0.1650	0.0762	0.0818

[a] $R_1 = \Sigma||F_o|-|F_c||/\Sigma|F_o|$. [b] $\omega R_2 = \{\Sigma[\omega(F_o^2-F_c^2)^2]/\Sigma[\omega(F_o^2)^2]\}^{1/2}$

high-resolution single-crystal X-ray diffraction data for complex **1**			
	70K	140 K	190 K
Formula	$C_{12}H_{14}CoN_8O_7$	$C_{12}H_{14}CoN_8O_7$	$C_{12}H_{14}CoN_8O_7$
Formula weight	441.24	441.24	441.24
Crystal system	Triclinic	Triclinic	Triclinic
Space group	*P*-1	*P*-1	*P*-1
a (Å)	8.2771(2)	10.3326(1)	8.2326(1)
b (Å)	10.2100(2)	10.7744(2)	10.0620(2)
c (Å)	10.6512(2)	15.3301(2)	10.9956(2)
α (deg)	88.019(2)	95.4470(10)	87.0770(10)
β (deg)	83.032(2)	102.0980(10)	84.1150(10)
γ (deg)	67.162(2)	93.5840(10)	68.244(2)
V (Å^3)	823.36(3)	1655.26(4)	841.42(3)
Z	2	4	2
D_{calcd} (g cm^{-3})	1.780	1.771	1.742
Wavelengths (Å)	0.4119	0.4119	0.4119
2Θ range for data collection (deg)	3.116-67.180	3.082-67.382	3.100-67.270
F(000)	450	900	450

R(int)	0.1414	0.1175	0.0662
GOF on F^2	1.086	1.041	1.097
R_1[a] [$I > 2\sigma(I)$]	0.0895	0.1275	0.0714
ωR_2[b](all data)	0.2343	0.3921	0.2319

[a] $R_1 = \Sigma||F_o|-|F_c||/\Sigma|F_o|$. [b] $\omega R_2 = \{\Sigma[\omega(F_o^2-F_c^2)^2]/\Sigma[\omega(F_o^2)^2]\}^{1/2}$

	complex **1-*d*14**		
	120 K	150 K	190 K
Formula	$C_{12}D_{14}CoN_8O_7$	$C_{12}D_{14}CoN_8O_7$	$C_{12}D_{14}CoN_8O_7$
Formula weight	453.26	453.26	453.26
Crystal system	Triclinic	Triclinic	Triclinic
Space group	*P*-1	*P*-1	*P*-1
a (Å)	8.2837(4)	10.3118(3)	8.2113(3)
b (Å)	10.1613(4)	10.7283(5)	10.0303(3)
c (Å)	10.6278(4)	15.3597(5)	10.9606(3)
α (deg)	88.064(3)	95.561(3)	87.119(2)
β (deg)	82.942(4)	101.988(3)	84.119(2)
γ (deg)	67.040(4)	94.061(3)	68.210(3)
V (Å^3)	817.37(6)	1647.10(11)	833.75(5)
Z	2	4	2
D_{calcd} (g cm^{-3})	1.850	1.836	1.814
F(000)	450	900	450
R(int)	0.0668	0.0743	0.0176
GOF on F^2	1.076	1.057	1.206
R_1[a] [$I > 2\sigma(I)$]	0.0688	0.0702	0.0290
ωR_2[b](all data)	0.1796	0.2065	0.0903

[a] $R_1 = \Sigma||F_o|-|F_c||/\Sigma|F_o|$. [b] $\omega R_2 = \{\Sigma[\omega(F_o^2-F_c^2)^2]/\Sigma[\omega(F_o^2)^2]\}^{1/2}$

Supplementary Table 2 Selected bond distances (Å) and angles (°) from the different phases of complex **1** and **1-d_{14}**.

Hp				
	SCXRD-1	SCXRD-1(hr)	SCXRD-1_d14	NPD-1_d14
Co1–O1W	2.0136(12)	2.0178(6)	2.0048(19)	2.0149(14)
Co1–O1	2.0981(11)	2.0968(6)	2.0900(16)	2.098(24)
Co1–O4	2.1352(11)	2.1348(6)	2.1304(17)	2.1350(16)
Co1–N1	2.1865(13)	2.1749(5)	2.181(2)	2.1871(13)
Co1–N3	2.1105(11)	2.1147(4)	2.1032(17)	2.1131(13)
Co1–N5	2.1748(12)	2.1924(6)	2.1706(18)	2.1770(15)
O1w–Co1–O1	93.28(5)	93.37(3)	93.38(8)	93.04(6)
O1w–Co1–N3	175.58(5)	175.44(3)	175.43(8)	173.58(7)
O1–Co1–N3	88.82(4)	88.64(2)	88.75(7)	89.32(6)
O1w–Co1–O4	90.16(5)	90.18(3)	90.05(8)	93.36(6)
O1–Co1–O4	169.91(5)	169.95(3)	170.04(7)	173.05(8)
N3–Co1–O4	87.13(4)	87.19(2)	87.31(6)	89.70(5)
O1w–Co1–N5	102.91(6)	110.28(4)	102.72(9)	99.90(6)
O1–Co1–N5	84.61(4)	97.09(3)	84.62(7)	85.19(7)
N3–Co1–N5	73.40(4)	73.48(2)	73.43(7)	74.56(5)
O4–Co1–N5	85.37(5)	90.48(3)	85.49(7)	87.92(6)
O1w–Co1–N1	109.84(6)	102.83(4)	109.87(9)	108.08(6)
O1–Co1–N1	97.31(5)	84.69(2)	97.42(7)	95.78(7)
N3–Co1–N1	73.70(5)	73.254(17)	73.81(7)	77.49(5)
O4–Co1–N1	90.42(5)	85.36(3)	90.25(8)	90.72(6)
N5–Co1–N1	146.99(5)	146.63(2)	147.12(7)	152.02(7)
Ip				
	SCXRD-1	SCXRD-1	SCXRD-1_d14	
Co1–O1W	2.0146(14)	2.0199(14)	2.0199(14)	
Co1–O1	2.1057(13)	2.1048(12)	2.1048(12)	
Co1–O4	2.1165(12)	2.1155(12)	2.1155(12)	
Co1–N1	2.2032(17)	2.2001(15)	2.2001(15)	
Co1–N3	2.1181(15)	2.1166(11)	2.1166(11)	
Co1–N5	2.1766(16)	2.1722(12)	2.1722(12)	
O1w–Co1–O1	91.93(6)	92.03(6)	92.03(6)	
O1w–Co1–N3	172.46(6)	172.52(7)	172.52(7)	
O1–Co1–N3	87.63(6)	87.58(5)	87.58(5)	
O1w–Co1–O4	93.22(6)	92.83(5)	92.83(5)	
O1–Co1–O4	171.85(5)	171.87(6)	171.87(6)	
N3–Co1–O4	86.44(6)	86.74(5)	86.74(5)	
O1w–Co1–N5	99.33(6)	99.18(6)	99.18(6)	
O1–Co1–N5	87.42(6)	87.34(5)	87.34(5)	
N3–Co1–N5	73.13(6)	73.23(4)	73.23(4)	

O4–Co1–N5	85.51(6)	85.45(6)	85.45(6)	
O1w–Co1–N1	113.82(6)	114.00(7)	114.00(7)	
O1–Co1–N1	94.35(6)	94.32(6)	94.32(6)	
N3–Co1–N1	73.72(6)	73.58(5)	73.58(5)	
O4–Co1–N1	89.35(6)	89.65(6)	89.65(6)	
N5–Co1–N1	146.69(6)	146.67(5)	146.67(5)	
Co2–O2	2.0344(14)	2.0338(13)	2.0338(13)	
Co2–O7	2.1073(13)	2.1048(12)	2.1048(12)	
Co2–O10	2.1092(12)	2.1051(13)	2.1051(13)	
Co2–N9	2.1597(17)	2.1618(14)	2.1618(14)	
Co2–N11	2.1133(15)	2.1041(14)	2.1041(14)	
Co2–N13	2.1646(17)	2.1621(15)	2.1621(15)	
O2w–Co2–O7	90.41(6)	90.61(6)	90.61(6)	
O2w–Co2–N11	174.97(6)	175.16(6)	175.16(6)	
O7–Co2–N11	86.62(6)	86.42(5)	86.42(5)	
O2w–Co2–O10	94.32(6)	94.09(6)	94.09(6)	
O7–Co2–O10	168.47(5)	168.60(6)	168.60(6)	
N11–Co2–O10	87.87(6)	88.15(6)	88.15(6)	
O2w–Co2–N13	102.05(6)	101.91(6)	101.91(6)	
O7–Co2–N13	85.07(6)	85.10(5)	85.10(5)	
N11–Co2–N13	73.65(7)	74.04(7)	74.04(7)	
O10–Co2–N13	83.75(6)	83.77(6)	83.77(6)	
O2w–Co2–N9	110.61(6)	110.09(6)	110.09(6)	
O7–Co2–N9	98.17(6)	97.94(6)	97.94(6)	
N11–Co2–N9	73.88(6)	74.16(6)	74.16(6)	
O10–Co2–N9	90.01(6)	90.19(7)	90.19(7)	
N13–Co2–N9	147.13(6)	147.79(6)	147.79(6)	
Lp				
	SCXRD-1	SCXRD-1	SCXRD-1_d14	NPD-1_d14
Co1–O1W	2.025(2)	2.0213(5)	2.0140(18)	2.0220(41)
Co1–O1	2.1214(19)	2.1216(6)	2.108(2)	2.1210(45)
Co1–O4	2.0994(19)	2.1034(6)	2.093(2)	2.0989(49)
Co1–N1	2.159(2)	2.1601(7)	2.159(2)	2.158(11)
Co1–N3	2.117(2)	2.1164(6)	2.101(2)	2.1036(22)
Co1–N5	2.159(2)	2.1609(6)	2.154(2)	2.1457(94)
O1w–Co1–O1	90.89(8)	90.98(2)	90.79(8)	88.21(5)
O1w–Co1–N3	171.77(8)	171.93(3)	172.12(8)	168.75(6)
O1–Co1–N3	84.08(8)	84.08(2)	84.46(8)	83.36(5)
O1w–Co1–O4	95.33(8)	95.16(3)	95.20(8)	98.53(8)
O1–Co1–O4	170.93(8)	170.88(2)	171.00(8)	173.26(7)
N3–Co1–O4	88.97(8)	89.03(2)	88.81(8)	89.94(5)
O1w–Co1–N5	99.80(8)	99.96(3)	100.11(8)	97.82(5)

O1–Co1–N5	88.85(8)	88.65(2)	88.51(8)	91.23(5)
N3–Co1–N5	73.66(9)	73.80(3)	73.55(9)	75.00(6)
O4–Co1–N5	83.59(8)	83.65(2)	83.84(8)	87.83(9)
O1w–Co1–N1	113.15(8)	113.10(3)	112.84(8)	112.04(8)
O1–Co1–N1	94.72(8)	94.72(3)	94.96(8)	90.93(12)
N3–Co1–N1	73.87(9)	73.58(3)	73.93(9)	75.66(5)
O4–Co1–N1	88.92(9)	89.08(3)	88.91(9)	86.59(6)
N5–Co1–N1	146.75(9)	146.66(2)	146.78(9)	150.12(13)

Supplementary Table 3 Bond distances (Å) and angles (°) of the potential hydrogen bonds in the HTp, Ip, and LTp structures of complex **1**.

D-H	∠DHA	*d*(D…A)	A
		HTp (190 K)	
C3-H3A	146.38	3.386	O1 [*x*+1, *y*, *z*]
C4-H4A	131.73	3.336	N7 [–*x*+2, –*y*+1, –*z*+1]
C4-H4A	148.87	3.38	O3 [–*x*+2, –*y*+1, –*z*+1]
C7-H7A	127.89	3.35	O6 [*x*, *y*-1, *z*]
C9-H9A	140.18	3.146	O4 [–*x*+1, –*y*+1, –*z*+2]
C9-H9A	166.15	3.369	O5 [–*x*+1, –*y*+1, –*z*+2]
C12-H12C	169.21	3.523	O6 [*x*–1, *y*, *z*]
O1W-H1WA	169.93	2.781	O3 [–*x*+1, –*y*+2, –*z*+1]
O1W-H1WB	127.66	3.246	N8
O1W-H1WB	127.56	3.136	O5 [–*x*+1, –*y*+2, –*z*+2]
O1W-H1WB	147.23	2.784	O6
		Ip (140 K)	
C3-H3	144.38	3.337	O7
C4-H4	136.31	3.298	N15 [–*x*+3, –*y*–1, –*z*]
C4-H4	157.34	3.455	O9 [–*x*+3, –*y*–1, –*z*]
C9-H9	139.47	3.15	O4 [–*x*+2, –*y*–2, –*z*–1]
C9-H9	165.62	3.383	O5 [–*x*+2, –*y*–2, –*z*–1]
C12-H12B	175.03	3.463	O12 [*x*–1, *y*, *z*–1]
C16-H16	165.99	3.501	N7 [–*x*+3, –*y*–1, –*z*]
C16-H16	165.5	3.41	O2 [–*x*+3, –*y*–1, –*z*]
C18-H18	144.92	3.487	O3 [–*x*+3, –*y*–1, –*z*]
C21-H21	127.17	3.044	O10 [–*x*+3, –*y*–2, –*z*]
C21-H21	147.96	3.31	O11 [–*x*+3, –*y*–2, –*z*]
O1W-H1WA	110.76	2.926	O3
O1W-H1WA	171.76	2.758	O9 [–*x*+2, –*y*–1, –*z*]
O1W-H1WB	117.27	2.914	O6
O1W-H1WB	154.2	2.888	O11 [–*x*+2, –*y*–2, –*z*]
O2W-H2WA	171.52	2.825	O3 [–*x*+2, –*y*–1, –*z*]
O2W-H2WA	113.91	3.043	O9
O2W-H2WB	160.41	2.811	O5 [–*x*+2, –*y*–2, –*z*]
O2W-H2WB	114.72	3.075	O12
		LTp (70 K)	
C1-H1B	129.98	3.293	O6
C3-H3A	149.42	3.299	O1 [*x*–1, *y*, *z*]
C4-H4A	165.68	3.505	N8 [–*x*+1, –*y*+1, –*z*+1]
C4-H4A	165.02	3.346	O2 [–*x*+1, –*y*+1, –*z*+1]
C6-H6A	146.27	3.479	O3 [–*x*+1, –*y*+1, –*z*+1]

C9-H9A	129.06	3.044	O4	[−x+2, −y+1, −z]
C9-H9A	145.94	3.399	O5	[−x+2, −y+1, −z]
C12-H12A	165.3	3.54	O5	[x+1, y, z]
O1W-H1WA	168.6	2.764	O5	[−x+2, −y, −z]
O1W-H1WB	111.5	3.014	O3	
O1W-H1WB	174.9	2.775	O3	[−x+2, −y, −z+1]

Supplementary Table 4 The structure parameters related to the orientation of the water and nitrates from the different phases of complex **1** and **1-d_{14}**.

		φ (°)	φ_1 (°)	φ_0 (°)	ψ (°)	ω (°)
HTp	SCXRD-1	85.30	27.56	0.65	133.53	159.64
	SCXRD-1(hr)	89.17	15.11	0.57	133.41	159.55
	SCXRD-1_d14	83.31	22.55	0.57	133.59	159.63
	NPD-1_d14	88.50	38.67	4.04	137.61	162.31
Ip	SCXRD-1	70.66 / 68.19	30.88 / 21.49	2.22 / 6.07	133.49 / 123.80	153.03 / 141.33
	SCXRD-1(hr)	75.01 / 58.35	58.75 / 25.92	2.13 / 6.07	133.53 / 123.93	153.32 / 141.23
	SCXRD-1_d14	71.79 / 69.48	29.97 / 20.77	2.30 / 6.12	134.04 / 123.28	151.45 / 140.85
	NPD-1_d14					
LTp	SCXRD-1	61.71	21.22	9.55	126.38	141.35
	SCXRD-1(hr)	68.00	15.17	9.31	126.47	141.24
	SCXRD-1_d14	67.70	22.78	9.03	126.22	141.43
	NPD-1_d14	67.73	36.26	12.19	129.51	141.75

φ, angle between the molecular plane and the plane of the coordinated water molecule;

φ_0, angle between the Co-O(water) bond and the molecular plane (defined by atoms N1, N3 and N5);

φ_1, angle between the Co-O(water) bond and the plane of the coordinated water molecule;

ψ, the dihedral angle N3–Co1–O1–O3;

ω, the dihedral angle N3–Co1–O4–O6.

Supplementary Table 5 Neutron powder diffraction Rietveld refinement results. The data represents **1-*d*$_{14}$** measured at 71 K and 194 K, where the unit cell parameters are as follows: a = 8.2580(14) Å, b = 10.2206(18) Å, c = 10.6350(12) Å, α = 88.0423(2)°, β = 83.0371(16)°, and γ = 67.1524(11)° with χ^2 = 18.3, R_{wp} = 3.79%, R_p = 2.79%, and R_e = 0.88% at 71 K; and a = 8.2349(17) Å, b = 10.0636(2) Å, c = 10.9946(17) Å, α = 87.0758(2)°, β = 84.0462(18)°, and γ = 68.2429(13)° with χ^2 = 9.83, R_{wp} = 3.11%, R_p = 2.28%, and R_e = 0.99% at 194 K.

**Name**	***x***	***y***	***z***	**B_{iso} (Å^2)**	**Occupancy**
			**71 K**		
Co1	0.9210(14)	0.2514(11)	0.2555(11)	1.56(2)	1
C1	0.6463(8)	0.0450(8)	0.3862(5)	B_{iso}(Co1)	1
D1C	0.5684(7)	0.0076(5)	0.3557(5)	1.5 B_{iso}(Co1)	1
D1B	0.7772(7)	0.0030(5)	0.3248(5)	1.5 B_{iso}(Co1)	1
D1A	0.6656(6)	0.0268(5)	0.4861(6)	1.5 B_{iso}(Co1)	1
C2	0.5598(8)	0.2020(8)	0.3735(5)	B_{iso}(Co1)	1
C3	0.3835(8)	0.2990(8)	0.4060(5)	B_{iso}(Co1)	1
D3A	0.2843(6)	0.2688(5)	0.4483(5)	1.5 B_{iso}(Co1)	1
C4	0.3777(10)	0.4306(8)	0.3790(5)	B_{iso}(Co1)	1
D4A	0.2637(7)	0.5305(6)	0.3877(4)	1.5 B_{iso}(Co1)	1
C5	0.5966(11)	0.5250(9)	0.2874(4)	B_{iso}(Co1)	1
C6	0.4953(8)	0.6697(9)	0.2830(5)	B_{iso}(Co1)	1
D6A	0.3513(7)	0.7134(6)	0.3068(5)	1.5 B_{iso}(Co1)	1
C7	0.7513(12)	0.7009(9)	0.1975(5)	B_{iso}(Co1)	1
D7A	0.8090(7)	0.7687(6)	0.1774(5)	1.5 B_{iso}(Co1)	1
C8	0.8511(9)	0.5560(9)	0.2016(4)	B_{iso}(Co1)	1
C9	1.1557(10)	0.5333(7)	0.1078(5)	B_{iso}(Co1)	1
D9A	1.1329(7)	0.6446(5)	0.1104(4)	1.5 B_{iso}(Co1)	1
C10	1.3000(9)	0.4171(9)	0.0678(5)	B_{iso}(Co1)	1
D10A	1.4302(7)	0.4226(5)	0.0202(5)	1.5 B_{iso}(Co1)	1
C11	1.2651(8)	0.2957(8)	0.1059(4)	B_{iso}(Co1)	1
C12	1.3740(9)	0.1417(8)	0.0833(5)	B_{iso}(Co1)	1
D12A	1.4698(6)	0.1526(5)	0.02570(5)	1.5 B_{iso}(Co1)	1
D12B	1.3953(6)	0.1018(5)	0.1715(6)	1.5 B_{iso}(Co1)	1
D12C	1.3110(6)	0.0770(5)	0.0313(4)	1.5 B_{iso}(Co1)	1
N1	0.6549(5)	0.2749(5)	0.3265(3)	B_{iso}(Co1)	1
N2	0.5448(8)	0.4151(5)	0.3317(4)	B_{iso}(Co1)	1
N3	0.7727(7)	0.4714(4)	0.2516(4)	B_{iso}(Co1)	1
N4	1.0333(6)	0.4850(5)	0.1673(4)	B_{iso}(Co1)	1
N5	1.1020(6)	0.3394(4)	0.1652(3)	B_{iso}(Co1)	1
N6	0.5772(7)	0.7542(4)	0.2361(4)	B_{iso}(Co1)	1
N7	0.9501(5)	0.2279(4)	0.5354(4)	B_{iso}(Co1)	1
N8	0.8143(4)	0.1389(4)	0.0247(4)	B_{iso}(Co1)	1
O1W	1.0981(8)	0.0512(7)	0.2748(5)	B_{iso}(Co1)	1
O1	0.9662(6)	0.2992(5)	0.4373(6)	B_{iso}(Co1)	1

O2	0.97849(6)	0.26618(5)	0.64431(6)	B_{iso}(Co1)	1
O4	0.85571(7)	0.22898(6)	0.075(5)	B_{iso}(Co1)	1
O3	0.88931(8)	0.1354(7)	0.52755(5)	B_{iso}(Co1)	1
O5	0.77023(7)	0.14993(6)	-0.09035(6)	B_{iso}(Co1)	1
O6	0.82374(6)	0.03831(6)	0.10347(5)	B_{iso}(Co1)	1
D1WA	1.11637(7)	-0.01168(5)	0.21114(4)	2.11(13)	1
D1WB	1.0973(6)	-0.02258(5)	0.35075(6)	B_{iso}(D1WA)	1
**194 K**					
Co1	0.5927(16)	0.7323(13)	0.7408(13)	1.16(6)	1
C1	0.9445(8)	0.8593(10)	0.5663(7)	2.76(6)	1
C2	0.9888(11)	0.7092(10)	0.6089(6)	B_{iso}(C1)	1
C3	1.1483(10)	0.5912(11)	0.5867(6)	B_{iso}(C1)	1
D3A	1.2586(10)	0.6048(9)	0.5453(7)	8.39(12)	1
C4	1.1181(11)	0.4737(10)	0.6330(7)	B_{iso}(C1)	1
D4A	1.2081(11)	0.3681(9)	0.6391(7)	B_{iso}(D3A)	1
C5	0.8545(13)	0.4361(11)	0.7329(6)	B_{iso}(C1)	1
C6	0.9305(10)	0.2894(11)	0.7592(6)	B_{iso}(C1)	1
D6A	1.0607(11)	0.2283(9)	0.7428(7)	B_{iso}(D3A)	1
C7	0.6556(12)	0.2966(11)	0.8450(6)	B_{iso}(C1)	1
D7A	0.5848(10)	0.2397(8)	0.8930(7)	B_{iso}(D3A)	1
C8	0.5850(11)	0.4391(12)	0.8206(6)	B_{iso}(C1)	1
C9	0.2789(13)	0.5067(8)	0.8963(6)	B_{iso}(C1)	1
D9A	0.2944(10)	0.4127(9)	0.9184(7)	B_{iso}(D3A)	1
C10	0.1405(10)	0.6325(9)	0.9143(6)	B_{iso}(C1)	1
D10A	0.0096(11)	0.6494(8)	0.9485(7)	B_{iso}(D3A)	1
C11	0.2030(10)	0.7417(9)	0.8765(5)	B_{iso}(C1)	1
C12	0.1250(9)	0.8953(10)	0.8695(7)	B_{iso}(C1)	1
N1	0.8689(6)	0.6669(6)	0.6703(3)	1.16(6)	1
N2	0.9455(8)	0.5203(6)	0.6790(4)	B_{iso}(N1)	1
N3	0.6869(9)	0.5084(4)	0.7699(4)	B_{iso}(N1)	1
N4	0.4169(8)	0.5409(6)	0.8437(4)	B_{iso}(N1)	1
N5	0.3694(6)	0.6864(6)	0.8283(3)	B_{iso}(N1)	1
N6	0.8318(7)	0.2224(4)	0.8164(4)	B_{iso}(N1)	1
N7	0.5034(5)	0.7639(4)	0.4717(5)	B_{iso}(N1)	1
N8	0.6852(5)	0.8578(6)	0.9698(4)	B_{iso}(N1)	1
O1	0.5020(6)	0.7042(6)	0.5762(7)	1.56(10)	1
O1W	0.4766(10)	0.9445(9)	0.7110(6)	B_{iso}(O1)	1
O2	0.4314(8)	0.7554(6)	0.3831(5)	B_{iso}(O1)	1
O3	0.5821(7)	0.8481(7)	0.4740(6)	B_{iso}(O1)	1
O4	0.6564(6)	0.7590(6)	0.9190(6)	B_{iso}(O1)	1
O5	0.7183(7)	0.8382(6)	1.0743(6)	B_{iso}(O1)	1
O6	0.6594(8)	0.9716(7)	0.9128(6)	B_{iso}(O1)	1
D1A	0.8618(10)	0.9310(8)	0.6451(8)	B_{iso}(D3A)	1
D1B	0.8572(11)	0.8914(7)	0.4961(7)	B_{iso}(D3A)	1

D1C	1.0567(11)	0.8738(7)	0.5548(6)	B_{iso}(D3A)	1
D12A	0.1633(10)	0.9485(8)	0.9386(8)	B_{iso}(D3A)	1
D12B	0.1445(8)	0.9454(7)	0.7743(8)	B_{iso}(D3A)	1
D12C	-0.0132(10)	0.9205(8)	0.8680(7)	B_{iso}(D3A)	1
D1WA	0.4785(9)	0.9964(8)	0.6400(7)	7.61(2)	1
D1WB	0.5122(10)	0.9859(8)	0.7707(7)	B_{iso}(D1WA)	1

Supplementary Table 6 Elements of the magnetic susceptibility tensor measured at 5 and 190 K (left) and their principal values (right).

T = 5 K and H = 1 kOe	
χ_{xx} = (1.23619 ± 0.00766)	
χ_{yy} = (1.19951 ± 0.00766)	
χ_{zz} = (2.71447 ± 0.00766)	
χ_{xy} = (0.49858 ± 0.00916)	χ_1 = 2.9277
χ_{yz} = (0.49662 ± 0.00916)	χ_2 = 1.5323
χ_{xz} = (0.18711 ± 0.00916)	χ_3 = 0.6901
T = 190 K and H = 5 kOe	
χ_{xx} = (2.94072 ± 0.00590)	
χ_{yy} = (2.10110 ± 0.00602)	
χ_{zz} = (3.12659 ± 0.00614)	
χ_{xy} = (0.65337 ± 0.00705)	χ_1 = 3.3050
χ_{yz} = (0.11736 ±0.00705)	χ_2 = 3.1358
χ_{xz} = (–0.10383 ±0.00705)	χ_3 = 1.7276

Supplementary Table 7 Principal components of the *g* tensor and the zero-field splitting parameters *D* and *E*.

	(S = 3/2)			(S = 1/2)			D (cm^{-1})	E (cm^{-1})
	g_x	g_y	g_z	g_x	g_y	g_z		
Exp. (HF-EPR)*	2.45	2.45	2.25	3.879	5.795	2.191	32.0	4.3
Calcd. (LTp)[a]	2.418	2.686	2.053	3.554	6.462	2.117	59.6	10.3
Calcd. (HTp)[a]	2.469	2.692	2.027	3.996	6.115	2.337	73.8	8.9
Calcd. (LTp)[b]	2.414	2.702	2.051	3.482	6.543	2.126	59.9	10.8
Calcd. (HTp)[b]	2.470	2.692	2.027	4.016	6.096	2.357	72.3	8.5
Calcd. (LTp)[c]	2.389	2.628	2.086	3.252	6.573	1.984	47.1	9.7
Calcd. (HTp)[c]	2.480	2.676	2.029	4.138	5.984	2.360	73.8	7.5
Calcd. (LTp)[d]	2.389	2.646	2.087	3.183	6.661	1.982	47.5	10.2
Calcd. (HTp)[d]	2.489	2.672	2.028	4.228	5.899	2.400	73.1	6.7
Calcd. (LTp)[e]	2.415	2.627	2.080	3.598	6.319	2.080	49.9	8.2
Calcd. (HTp)[e]	2.481	2.681	2.028	4.129	5.996	2.346	73.4	7.7
Calcd. (LTp)[f]	2.438	2.647	2.083	3.727	6.277	2.138	53.4	8.1
Calcd. (HTp)[f]	2.500	2.688	2.031	4.249	5.915	2.412	76.7	7.1
Calcd. (LTp)[g]	2.400	2.632	2.084	3.378	6.490	2.021	48.4	9.2

* Simultaneous fitting of the HF-EPR and magnetic susceptibility. g_x and g_y are restricted to be the same.

[a] Calculation based on the H-optimized geometry and active space constructed from the five 3d orbitals.

[b] Calculation based on the H-optimized geometry and active space constructed from the five 3d orbitals and five 4d orbitals as a double shell.

[c] Calculation based on the H-fixed (according to the typical bond lengths obtained from neutron diffraction measurements and the X-ray determined bond angles) geometry and active space constructed from the five 3d orbitals.

[d] Calculation based on the H-fixed (according to the typical bond lengths obtained from the neutron diffraction measurements and the X-ray determined bond angles) geometry and active space constructed from the five d orbitals and five 4d orbitals as a double shell.

[e] Calculation based on the H-optimized geometry including two nitric acid forming H-bonds with water molecule and active space constructed from the five 3d orbitals.

[f] Calculation based on the H-optimized geometry and active space constructed from the five 3d orbitals and their bonding partner orbitals.

[g] Calculation based on the H-optimized geometry including two neighboring complex motifs forming H-bonds with water molecule and active space constructed from the five 3d orbitals.

Supplementary References

1 Elhaïk, J. *et al.* The spin-states and spin-crossover behaviour of iron(II) complexes of 2,6-dipyrazol-1-ylpyrazine derivatives *Dalton Trans.* 2053-2060 (2003).

2 Sawama, Y., Monguchi, Y. & Sajiki, H. Efficient H–D Exchange Reactions Using heterogeneous platinum-group metal on carbon–H_2–D_2O system. *Synlett* **23**, 959-972 (2012).

3 Chilton, N. F., Anderson, R. P., Turner, L. D., Soncini, A. & Murray, K. S. PHI: A powerful new program for the analysis of anisotropic monomeric and exchange-coupled polynuclear d- and f-block complexes. *J. Comput. Chem.* **34**, 1164-1175 (2013).
