# Supplementary material for: Water-oriented magnetic anisotropy transition
Source: Nat Commun. 2021 May 12;12:2738. doi: 10.1038/s41467-021-23057-4 (PMC8115317; doi:10.1038/s41467-021-23057-4)
Supplement: Supplementary file 3 — Description of Additional Supplementary Files [file 41467_2021_23057_MOESM3_ESM.pdf]

Description of Additional Supplementary Files

Supplementary Data 1 The atomic coordinates of optimized models of HTp and LTp
